# The experience of a regional network of health policy and systems actors in translating evidence into policy and action in West Africa

**DOI:** 10.4314/gmj.v56i3s.11

**Published:** 2022-09

**Authors:** Ermel A K Johnson, Ijeoma N Okedo-Alex, Sitsofe Gbogbo, Arnold I Okpani, Selina Defor, Felix A Obi, Jean-Paul Dossou, Ejemai Eboreme

**Affiliations:** 1 West African Network of Emerging Leaders for Health Policy and Systems Research (WANEL), Accra, Ghana; 2 African Institute of Health Policy and Health Systems, Ebonyi State University, Abakaliki, Nigeria; 3 West African Health Organisation (WAHO), Bobo Dioulasso, Burkina Faso; 4 University of Health and Allied Sciences, Ho, Ghana; 5 National Primary Health Care Development Agency, Abuja, Nigeria; 6 Dodowa Health Research Centre-Ghana Health Service, Dodowa, Ghana; 7 Results for Development (R4D) Nigeria Country Office, Abuja, Nigeria; 8 Centre de recherche en Reproduction Humaine et en Démographie, Cotonou, Benin; 9 National Primary Health Care Development Agency, Abuja, Nigeria; 10 Department of Psychiatry, Faculty of Medicine and Dentistry, University of Alberta, Alberta, Canada

**Keywords:** Knowledge Translation, Health Policy, West Africa

## Abstract

**Objectives:**

To identify strategies and interventions to strengthen the generation and use of research evidence in health policy and practice decision-making and implementation in the West African sub-region (knowledge translation).

**Design:**

The study design was cross-sectional. Data sources were from a desk review, West African Network of Emerging Leaders (WANEL) member brainstorming, and group discussion outputs from WANEL members and session participants' discussions and reflections during an organised session at the 2019 African Health Economics and Policy Association meeting in Accra.

**Results:**

Strategies and interventions identified included developing a Community of Practice, a repository of health policy and systems research (HPSR) evidence, stakeholder mapping, and engagement for action, advocacy, and partnership. Approaches for improving evidence uptake beyond traditional knowledge translation activities included the use of cultural considerations in presenting research results and mentoring younger people, the presentation of results in the form of solutions to political problems for decision-makers, and the use of research results as advocacy tools by civil society organisations. Development of skills in stakeholder mapping, advocacy, effective presentation of research results, leadership skills, networking, and network analysis for researchers was also identified as important.

**Conclusions:**

To strengthen the generation and use of research evidence in health policy and practice decision-making in West Africa requires capacity building and multiple interventions targeted synergistically at researchers, decision-makers, and practitioners.

**Funding:**

Funding for the study was provided by the COMPCAHSS project (#108237) supported by IDRC

## Introduction

### Status of Evidence-Informed Decision-Making in West Africa

The importance of using scientific evidence in decision-making and health practice is recognised and well-documented in the literature.^1^–^3^ However, its systematic use by policymakers is far from established in West Africa.^4^–^5^ Although several initiatives have been implemented in the sub-region to promote the use of evidence in policy and practice 6–9, they have had limited success. While some policy or strategy documents are developed based on previous policy evaluation reports or feasibility study results, neither research evidence nor stakeholder input – termed “informal evidence”- are utilised systematically and transparently.^10^

In West Africa, the level of use of evidence in decision-making varies according to the context and especially according to the level in the health system. English-speaking countries have a certain advance over other French-speaking and Portuguese-speaking countries, as the concepts of evidence-informed decision-making (EIDM) were developed and popularised in English. The difficulty of translating certain concepts and terminology used in the field of EIDM also constitutes a barrier.^11^

The generation, translation, and use of evidence, whatever the nature and origin, requires capacity^12^. This capacity ranges from accessing, analysing, synthesising, adapting, using, and evaluating, all of which vary according to the stages of the policymaking process^13^. Each stage of the evidence-use process requires particulars knowledge and skills. But despite recent advancements in knowledge translation (KT) and implementation science, a recent assessment revealed that current capacity-building programs are inadequate to meet the need in low-and middle-income countries (LMICs).^14^ Consequently, the knowledge and skills for evidence generation, translation, and use by actors involved in decision-making are low.^15^,^16^ Knowledge brokers and other knowledge translation services supplement these capacities, making relevant and timely evidence available to decision-makers throughout the health policy and decision-making cycle. The experience of the West African Health Organisation (WAHO) in playing the role of Health Policy and Research Organisation (HPRO) in promoting the use of evidence in policy and practice has shown the need for a regional body or structure to serve as a link between the production and use of evidence in policy and practice.^17^–^20^ As reported by Agyepong et al., researchers need to be exposed to decision-making fora and platforms to build collaborative relationships with decision-makers, identify research needs, and have a good understanding of policymaking strategies and context particularities.^21^

### WANEL, as a springboard for EIDM in West Africa

The West African Network of Emerging Leaders (WANEL) for Health Policy and Systems Research (HPRS) is a network born out of the desire of pioneers in the field of HPRS in West Africa to contribute to the strengthening of health systems through health systems research and to promote this field of research, which is still little known in the sub-region. Thus, since 2015, WANEL has brought together actors from various fields related to health systems. The network presents itself as a niche for health systems strengthening through health systems research. Members of the network are mid-level managers in research institutions or organisations operating in the sub-region and are present in the fifteen countries of the Economic Community of West African States (ECOWAS).^22^

The members are active professionals in diverse and complementary disciplines and include epidemiologists, sociologists, journalists, university teachers, programme and project managers. Members contribute through research in their respective fields to the production and dissemination of knowledge and information for improving the health system. Thus, WANEL presents itself as a technical tool that can serve and contribute to developing the capacity to generate and promote the use of evidence in health policy and practice across the sub-region.^22^

This article is an output of one of WANEL's objectives, which is to identify KT strategies and interventions and develop a framework for the generation, translation, and use of evidence in policy and practice in the West African sub-region.

## Methods

### Study scope and design

The study was conducted as part of WANEL's structuring and formalisation process. Four thematic groups were set up to ensure the implementation of interventions that would enable WANEL to achieve its mission. A participatory study of WANEL members was initiated by the thematic group on “Generating and Translating Evidence into Policy” to develop the group's knowledge translation (KT) framework for creating and promoting the use of research findings in policy and practice in West Africa. The group used the opportunity of the African Health Economics Association (AfHEA) 2019 conference in Accra to hold an organised session where members and other participants were invited to reflect on strategies and capacities needed to influence policy and practice through research evidence.

### Data collection and analysis

Data collection was conducted in two phases. *Phase 1*: Documents review: In the first phase, data were collected from WANEL's core documents, including WANEL's mission statement and the terms of reference of the thematic group “Generating and Translating Evidence into Policy”.

*Phase 2*: Discussions in the context of an organised scientific session at the African Health Economics and Policy conference in Accra 11–14 March 2019. The two-hour session involved thirty persons made up of WANEL members and other participants from various countries in the sub-region. Representation by country of origin of the participants is summarised in Table 1.

**Table 1:** Country origin of participants

Country	Number of persons
**Bénin**	2
**Burkina Faso**	3
**Côte d'Ivoire**	1
**The Gambia**	1
**Ghana**	12
**Guinea**	1
**Nigeria**	9
**Senegal**	1
**Total**	30

Three case study abstracts presenting preliminary results of ongoing research by WANEL members had been accepted by the AfHEA conference for oral presentation as part of this WANEL session. Box 1 summarises the topics of the studies presented. The presentation of the evidence from the three research projects was used as the entry point for discussions on how the uptake of research evidence can be strengthened in the West African sub-region.

**Box 1** Ongoing case studies presented during the organised session

1. Adolescent mothers want easy access to antenatal care services in the Hohoe Municipality of Ghana: Findings from a phenomenon research
2. A Participatory Action Research for health system bottleneck analyses in a Prevention of Maternal to Child Transmission of HIV programme in Nigeria
3. The midwives service scheme: a qualitative comparison of contextual determinants of the performance of two states in central Nigeria

The general discussion that followed the presentations of the research findings used a fish-bowl exercise format. This approach is well documented in the literature as an effective method for co-creation.^23^,^24^ It consisted of asking four questions that applied to the presentations made to facilitate discussion about how to influence policy and practice with the results presented. Box 2 presents the questions that guided the fish-bowl session.

**Box 2** Questions discussed in the group

1. How can the evidence from the case studies improve maternal and child health (MCH) outcomes in West Africa?
2. Which KT interventions in MCH contribute to translating the research into policy and practice in West Africa?
3. Which strategies should WANEL use to translate evidence in policy and action in West Africa?
4. Which capacities are needed, and how to strengthen them?

Field notes and meeting reports from the organised session were collated for analysis. The data collected were triangulated through informal discussions with the case study presenters and participants in the discussion session. The triangulation provided a good understanding of the data collected from the documents, reports and notes taken during the organised session. Two researchers (EAKJ and EE) coded the data collected and then conducted an inductive thematic analysis.

### Ethical approval

This research presented in the three presentations was conducted with ethical clearance. The presentation and discussion of the results and strategies for research uptake of these and similar results did not collect any personal information from participants. Participants attended freely in the organised session and were not forced to participate in the discussion. All information compiled during the organised session was public. The individual discussions undertaken with the presenters were only related to the presentations made. WANEL activities are part of the Consortium for maternal, child, adolescent health policy and systems strengthening (COMCAHSS) project funded by IDRC. The midterm review of the program and its interventions and outputs received ethical clearance from the Ghana Health Service Ethical Review Committee in November 2018 (GHS-ERC 012/10/18).

## Results

The focus of this paper is on the discussion of the development of appropriate Knowledge translation (KT) interventions, strategies, and capacities for research to inform WANEL proposals and interventions for KT rather than the content of the three studies presented in the organised session and used as an entry point for the discussion. Our presentation of results, therefore, focuses on this objective and not the content of the three research projects.

### Knowledge Translation strategies

Six key strategies were identified to promote the use of evidence in policy and practice: *i)* Establish a repository of evidence generated from research conducted by network members or other important studies conducted in the sub-region. *ii)* Development of stakeholder mapping that could contribute to health systems strengthening in the sub-region and that could influence health policies. *iii)* Formation of communities of practice through the different thematic working groups or through addressing any other topic that may be necessary and appropriate. *iv)* Stakeholders' commitment to action. *v)* Advocacy and lobbying through the WANEL ambassador to promote the network in regional spaces to create linkages; and finally, *vi)* Partnership with different sub-regional institutions and bodies thereby enabling WANEL to become members or stakeholders of these institutions. A summary of the strategies identified is presented in Table 2. Examples of the application of these strategies have been listed to illustrate efforts already undertaken.

**Table 2:** Knowledge Translation strategies identified

KT strategies	Examples of application
Evidence repository	In 2019, the repository of WANEL members' scientific output included 70 published articles.
Stakeholder mapping	The main institutions at the regional level and their areas of intervention were identified, which could serve as a niche for the network, with a focus on WAHO.
Community of Practice	Creation of various thematic groups, including “Generating and Translating Evidence into Policy”
Stakeholder engagement for action	In February 2017 in Niamey, WANEL had a strategic meeting with the Director-General of WAHO to present the competencies and skills to be made available to the institution and the possible support areas. In 2019, several meetings were held with the Chair of AfHEA to present the competencies and skills of WANEL members that could serve AfHEA.
Advocacy and Lobbying	An Ambassador of the network was appointed to act as a link in regional decision-making bodies and to do advocacy
Partnership	WANEL has established a working partnership with the non-governmental organisation Initiative Prospective Agricole et Rurale (IPAR) on health-related SDG indicators in the sub-region. WANEL was officially admitted as a member of the AfHEA board

### Knowledge Translation interventions

The strategies identified must be translated into action. In addition to the usual knowledge translation interventions, more specific interventions were proposed. The list of these interventions and examples of proposed applications are presented in Table 3.

**Table 3:** Knowledge Translation interventions identified

KT interventions	Examples/proposals for application
Problem-solving presentation of evidence	Presenting research evidence in the manner of resolving burning issues faced by policymakers, NGOs, CSOs in the region
Community outreach for behaviour change	Developing docudrama and cartoons on health issues targeting communities to promote behaviour change
Provision of evidence to CSOs as an advocacy tool	Developing issues briefs, infographics, and compelling stories for more influential CSOs active in health in the sub-region.
Cultural consideration in evidence presentation	Using adolescent health as an example, make interventions at the level of families, communities, and schools and consider social and cultural constraints.
WANEL Conferences	Organising regional conferences to share health policy and systems research evidence, create policymakers-research collaboration discussion on priorities and agenda for health policy and system
Assisting regional institutions in achieving their mission	Developing Memoranda of Understanding (MoU) and collaboration agreements with regional bodies to produce research evidence in support of achieving their missions

The interventions reflect outside-the-box thinking and are aimed at various levels, from field actors such as NGOs, CSOs, and the community to regional institutions. The evidence must therefore be presented in a format that addresses the issues encountered in day-to-day activities. At each level of the health system and for each group of actors, the interventions to be implemented will be adapted and blended to promote a synergy of action.

### Capacities required

Skills must enable WANEL members to identify strategies and implement interventions. The core skills needed for the transfer of research findings into policy and action were advocacy, strategic communication, presenting research findings in attractive and actionable ways, working with decision-makers, stakeholder mapping, stakeholder engagement, leadership, networking and network analysis, and understanding policy formulation processes in the ECOWAS context. In addition to these cross-cutting competencies, capacity-building in specific technical areas, such as cost analysis of interventions and systems thinking, was proposed. Language skills, especially French, English and Portuguese, are key to allowing the full contribution of all network members.

For developing these capacities and skills, summer schools, short courses, practical training workshops, and internships in specialised institutions with mentors could be opportunities to explore. As well, a language exchange between network members for linguistic skills immersion in the desired language could be easily organised and inexpensive.

### WANEL KT framework: generating and translating evidence into policy

**Figure 1:**
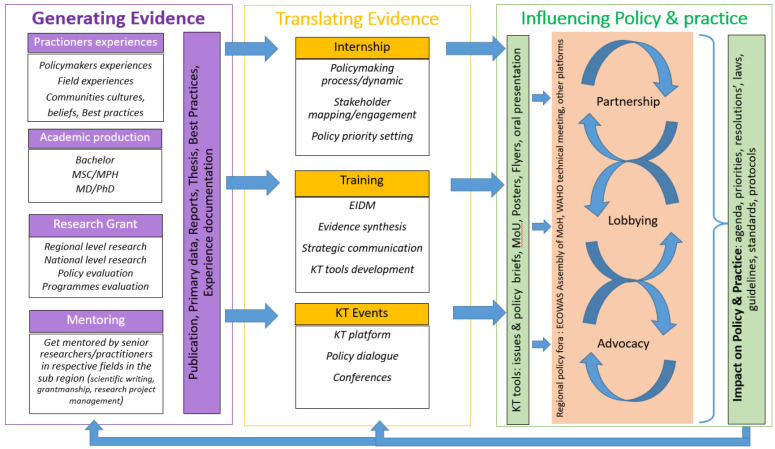
WANEL Knowledge translation framework

The synthesis of the results of the data analysis enabled the development of a conceptual framework for knowledge translation that informs the work of the WANEL thematic group on KT or the WANEL Knowledge translation framework (figure 1). The framework reflects a dual focus on evidence production and evidence transfer into policy. Evidence transfer into policy is subdivided into evidence transfer and influencing policy and practice. Thus, the conceptual framework is broadly divided into three axes: *Generating Evidence, Translating Evidence*, and *Influencing Policy & Practice*. Each axis includes strategies and interventions to be implemented. The knowledge production axis *“Generating Evidence*” comprises three strategies (academic production, conducting research projects, and mentoring) for which specific interventions have been identified. These different interventions will make it possible to produce knowledge, data, and innovations that will feed into the *Translating Evidence* axis. This second axis includes three strategies: i) internships in public and private political institutions and organisations, ii) specific capacity-building and iii) knowledge translation events. For each strategy, interventions have been identified to provide members with the skills required to put the research results, knowledge, and innovations from the “*Generating Evidence*” axis into formats adapted to the beneficiaries, using appropriate knowledge translation tools and support. The knowledge translation products from this axis will be used in the third axis of *Influencing Policy & Practice*. In this last axis, the tools to be used are, among others, the knowledge translation products from the *“Translating Evidence*” axis, as well as partnership, collaboration and consultancy agreements.

The strategies identified in this axis are advocacy, lobbying, and partnership with regional and international organisations and institutions working in the sub-region. These strategies for influencing policies will occur in different spaces and existing decision-making fora in the sub-region, ranging from technical meetings for developing strategic documents to the ECOWAS Assembly of Ministers of Health. The framework aims to impact health policy agendas, priorities, and practices (choice of interventions, equity, gender, quality of care, and organisation of health services). A mechanism for systematic monitoring, evaluation, and documentation of impact will guide the new knowledge to be generated and research to be conducted as well as the synthesis of knowledge to better meet the needs of stakeholders and to have a greater impact on policy and practice.

## Discussion

Our study has enabled us to generate a conceptual frame-work to inform knowledge translation efforts in the West African sub-region across the spectrum of generating, translating, and influencing policy and practice, as depicted in the WANEL KT framework. The framework is a guide for capacity building, advocacy and policy planning activities that informs the WANEL network but is also of potential relevance to other sub-regional health research and policy networks beyond the West African sub-region.

### The feasibility of the identified strategies and interventions.

The process of using evidence in policy and practice begins with the accessibility of evidence. This accessibility consists of the production of research evidence on health system problems that propose contextually appropriate solutions, which must be disseminated. The value of an online directory at the sub-regional level lies in the difficulty of obtaining information on policy documents, strategy documents, and other official country documents. These documents are mostly scattered across departments and institutions, making them difficult to access. The availability of a repository of research and other health data from countries in the sub-region would increase the accessibility, especially through online resources. This online evidence repository contributes to increasing the impact of research findings, supporting learning for researchers, and providing sources of new research ideas.^25^–^27^ Most of the available directories are global with no adaptation to the contexts of the West African sub-region except for the Ebonyi State University Policy Information Platform, which focuses on Nigeria.^28^ The type and format of information available in the directories are also factors of attractiveness for use 27; while the ease of use of the platforms and the quality of the documents are elements of non-use.^28^

WANEL members produce information or evidence from research or work that can strengthen health systems in the sub-region. However, these products are not catalogued in an easily accessible place; hence, the importance of developing a network directory.

Stakeholder mapping and engagement for action are important strategies for regional interventions. It is important to know which major actors can influence the health policies and systems of the countries in the sub-region. A good understanding of their interests and areas of influence would enable the development of specific interventions for them to use the evidence to strengthen health systems. This informed WANEL's choice of this strategy for its development, accessing regional institutions and bodies and providing them with the necessary evidence for health systems strengthening. The development of partnerships with identified stakeholders, the development of advocacy actions, and communities of practice contribute to the strengthening of stakeholder engagement and recognition.

### Opportunities for promoting KT for health systems and policy strengthening in West Africa

Orton et al., in a systematic review, concluded that there is an urgent need to generate knowledge to support the use of evidence in public health policies.^29^ The field of health policy and systems research is not well developed in the sub-region, nor is the use of evidence in health policy. There is an important need for capacity building of actors (researchers, policy makers and practitioners), improvement of the working environment for an effective application of strategies promoting the use of evidence in health policies and practice. Indeed, regional institutions such as the West African Health Organisation, the African Population Health Research Centre have experimented with a model of promoting and supporting the use of evidence through the role of the HPRO.^30^,^31^ The readiness and willingness of regional research and policy institutions to promote the use of evidence in policy and practice provide an opportunity for future KT interventions in Africa. In addition, researchers and policy makers are increasingly aware that evidence in health policy and practice may be receptive to capacity-building programmes and KT interventions. Also, evidence promotion networks such as WANEL, and Africa Evidence Network, through their KT activities, constitute opportunities for evidence promotion that can contribute to health systems strengthening. Funding of research initiatives for health systems strengthening through implementation research by donors such as IDRC also offers opportunities.^30^,^32^

### Limitations to the application of the WANEL frame-work and KT strategies

Despite the identification of these opportunities and the development of this framework, limitations to their application exist. The first is that the effectiveness of these strategies is dependent on the assumption of willingness to implement evidence-based practice by knowledge users and policymakers. Another is the availability of resources (human, financial, and time). These limitations are well documented in the literature^33^–^37^. Further, the change resulting from most KT strategies is gradual, and impact may be seen in the long term^12^.

## Conclusion

Health systems strengthening in the West African sub-region, and indeed globally depends on several factors, including the use of research evidence in public policy. Conceptual frameworks, strategies, and interventions to inform the strengthening of capacities and use of research evidence in policy and practice adapted to the realities of varying contexts are urgently needed. The West African Network of Emerging leaders (WANEL) in Health Policy and Systems Research has developed the conceptual framework presented in this paper from their work in West Africa. Applying this KT framework and its evaluation over time and adaptation to context as relevant is an important contribution to the field of work known as Knowledge Translation or Research Uptake in West Africa and beyond.
